# Surveillance of SARS-CoV-2 immunogenicity: loss of immunodominant HLA-A*02-restricted epitopes that activate CD8^+^ T cells

**DOI:** 10.3389/fimmu.2023.1229712

**Published:** 2023-11-03

**Authors:** Ágata Lopes-Ribeiro, Patrícia de Melo Oliveira, Henrique Morais Retes, Edel Figueiredo Barbosa-Stancioli, Flávio Guimarães da Fonseca, Moriya Tsuji, Jordana Grazziela Alves Coelho-dos-Reis

**Affiliations:** ^1^ Laboratório de Virologia Básica e Aplicada, Instituto de Ciências Biológicas, Departamento de Microbiologia, Universidade Federal de Minas Gerais, Belo Horizonte, Brazil; ^2^ Centro de Tecnologia (CT) Vacinas, Universidade Federal de Minas Gerais, Belo Horizonte, Brazil; ^3^ Aaron Diamond AIDS Research Center, Irving Medical School, Columbia University, New York, NY, United States

**Keywords:** CD8^+^ T cells, SARS-CoV-2, HLA-A2, peptide microarray, immunoinformatics

## Abstract

**Introduction and methods:**

In this present work, coronavirus subfamilies and SARS-CoV-2 Variants of Concern (VOCs) were investigated for the presence of MHC-I immunodominant viral peptides using in silico and *in vitro* tools.

**Results:**

In our results, HLA-A*02 haplotype showed the highest number of immunodominant epitopes but with the lowest combined prediction score. Furthermore, a decrease in combined prediction score was observed for HLA-A*02-restricted epitopes when the original strain was compared to the VOCs, indicating that the mutations on the VOCs are promoting escape from HLA-A2-mediated antigen presentation, which characterizes a immune evasion process. Additionally, epitope signature analysis revealed major immunogenic peptide loss for structural (S) and non-structural (ORF8) proteins of VOCs in comparison to the Wuhan sequence.

**Discussion:**

These results may indicate that the antiviral CD8^+^ T-cell responses generated by original strains could not be sufficient for clearance of variants in either newly or reinfection with SARS-CoV-2. In contrast, N epitopes remain the most conserved and reactive peptides across SARS-CoV-2 VOCs. Overall, our data could contribute to the rational design and development of new vaccinal platforms to induce a broad cellular CD8^+^ T cell antiviral response, aiming at controlling viral transmission of future SARS-CoV-2 variants.

## 1 Introduction

The SARS-CoV-2 pandemic is associated with the coronavirus disease-2019 (COVID-19), responsible for the increase in hospitalizations for pneumonia with possible development to multiple organ failure. The first manifestations of COVID-19 were observed in December 2019, but since then, SARS-CoV-2 has resulted in over 670 million identified cases and 6.8 million confirmed deaths worldwide (1). In December 2020, several countries started immunization programs against SARS-CoV-2, totalizing over 13 billion doses of vaccines administrated by 2023 (1). However, despite many efforts to increase vaccinal coverage, only 69% of the world population received at least one dose of these immunizers. Moreover, such rates are heterogeneous and may reach under 30% in low-income regions (2). Overall, approximately 5.7 million new cases of COVID-19 were reported at the beginning of 2023 (1).

Considering the continuous observations of long-term sequels even in individuals with mild cases of the disease (3), the number of new cases that continue to arise daily brings a new and worrying aspect to the SARS-CoV-2 pandemic. This scenario is further aggravated by the spread of new variants and subvariants, with higher rates of transmission and mutations that lead to the immune escape of antibody response (4, 5) generated by vaccination. In addition to the humoral response, T-cell responses also play a critical role in antiviral protection against SARS-CoV-2 and are usually reported as relatively less susceptible to immune evasion than the antibody response (6), however, considering the rapid-paced evolution of SARS-CoV-2, these considerations must be taken with care. The evaluation of T cells from individuals convalescing for COVID-19 against stimulation with different SARS-CoV-2 peptides, for example, indicated the persistence of a robust cellular response, strongly characterized by the CD8^+^ T lymphocyte compartment (7). However, while CD8^+^ T cells showed greater reactivity in individuals recovered from mild cases of the disease, the response of CD4^+^ T cells was predominant in individuals who had severe COVID-19, indicating possible protective role for CD8^+^ T cells (7).

Indeed, the correlation between the early establishment of the CD8^+^ T cell response and mild cases of COVID-19 is well established (8), and the protective action of these cells against SARS-CoV-2 infection has already been described, including for individuals with significant disturbances in the humoral response (9). Not only that, but viral clearance is largely dependent on cytotoxic CD8^+^ T lymphocyte responses (10, 11). Therefore, it is paramount to take CD8^+^ T cell subsets as an asset in inducing immunogenicity and long-lasting memory responses against SARS-CoV-2.

Long-lasting memory CD8^+^ T cell responses are developed upon activation of 9-15 amino acid viral peptide sequences presented by human Major Histocompatibility Complex (MHC) or Human Leukocyte Antigen (HLA) in humans (12, 13). Peptide-MHC interactions are able to activate T-cell receptors (TCR) generating diverse antiviral T-cell repertoire (14, 15). It is still unknown whether the anti-SARS-CoV-2-specific clones of CD8^+^ T cell responses induced by either vaccination and/or disease associated to the previous variants of concern would be efficacious to the new variants of concern emerging in a fast pace and real-time. In fact, the T-cell COVID-19 Atlas (T-CoV, https://t-cov.hse.ru) provides a comprehensive web portal, which allows the in silico analysis of SARS-CoV-2 mutations and how they can alter the presentation of viral peptides by HLA molecules (16). In this sense, although comprehensive in silico tools are available, there is still a lack of CD8 T cell epitope mapping using *in vitro* methods for confirming these robust in silico results.

Hence, in the present study, in silico and *in vitro* techniques were combined to evaluate HLA-A*02-restricted coronavirus peptide signatures with a focus on SARS-CoV-2. This comprehensive overview emphasized the occurrence of CTL (cytotoxic T lymphocytes) epitope loss for structural and non-structural proteins in SARS-CoV-2 variants in comparison to the original Wuhan strain, bringing new insights regarding the antiviral CD8^+^ T-cell response against SARS-CoV-2. The current study will contribute to the development of new vaccinal platforms to induce a broad cellular antiviral response, aiming to control viral transmission.

## 2 Materials and methods

### 2.1 Sequence data collection and peptide prediction

**Figure 1:**
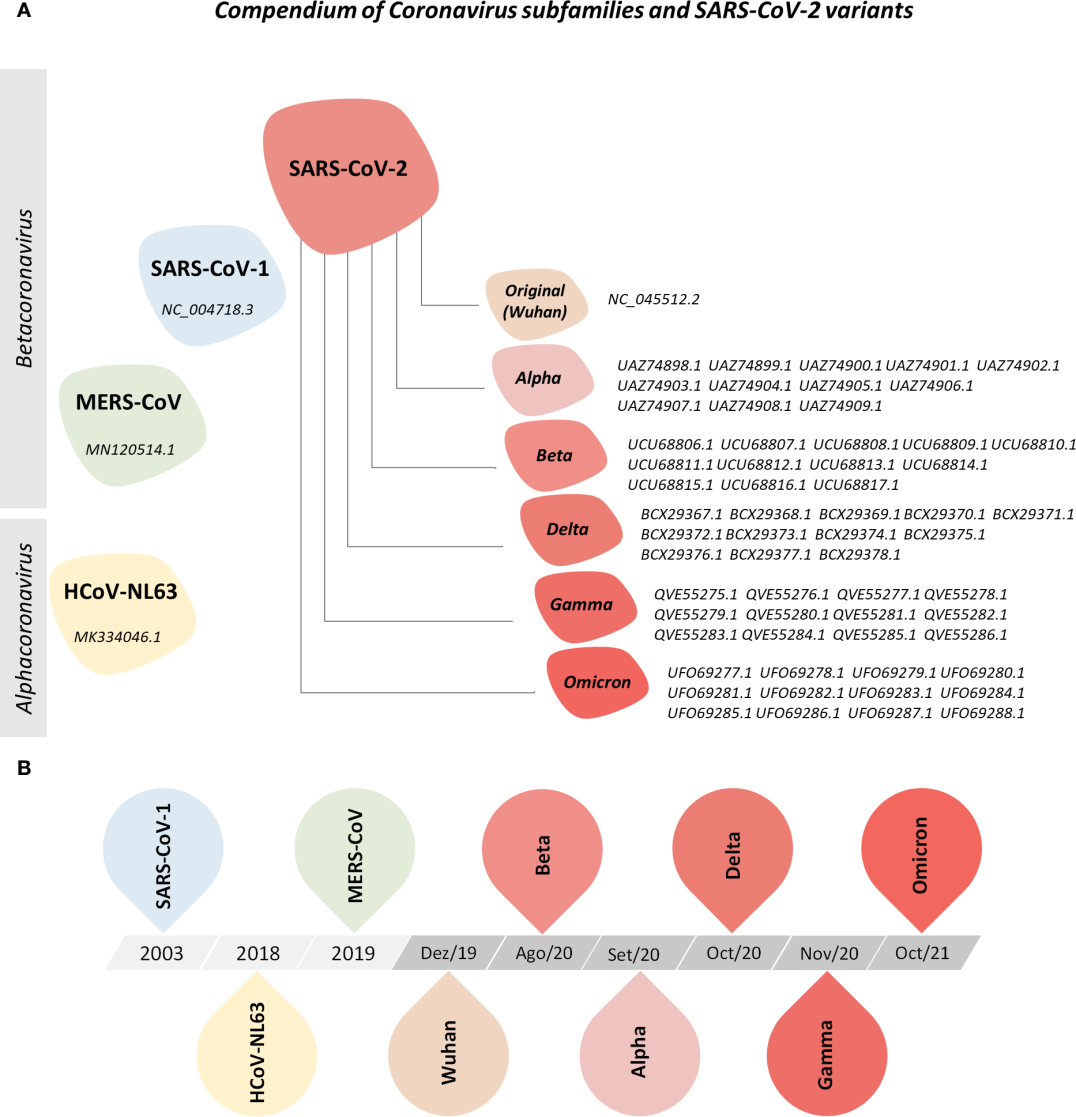
Compendium of Coronavirus Subfamilies and SARS-CoV-2 Variants. **(A)** Overview of human alphacoronavirus HCoV-NL63 and betacoronavirus MERS-CoV, SARS-CoV and SARS-CoV-2 (Wuhan, Alpha, Beta, Delta, Gamma, and Omicron variants) included in this study. All protein sequences were collected from the NCBI nucleotide and NCBI SARS-CoV-2 database. **(B)** Timeline of sequence deposit on NCBI nucleotide database.

For this study, protein reference sequences from HCoV-NL63, MERS-CoV, SARS-CoV-1, and SARS-CoV-2 and from five variants of concern (VOCs) from SARS-CoV-2 were acquired from the NCBI public database (**Supplementary Tables 1**–**3**). **Figure 1** illustrates the universe of coronaviruses studied here.

Acquired sequences were applied for the prediction of 9-mer peptides in NetCTL 1.2 software (https://services.healthtech.dtu.dk/service.php?NetCTL-1.2/), using peptide-MHC-I affinity, peptide cleavage and transport by the transporter associated with antigen protein complex (TAP) to determine an overall combined score (CS) (17). Peptides with a CS ≥0.75 are presumed to be immunogenic for CD8^+^ T cells and kept for the evaluation of prediction parameters as well as for in silico prediction of cytokine induction and biochemical parameters.

Peptide prediction was performed for six of the most prevalent MHC-I haplotypes in the Brazilian population, namely supertypes HLA-A*01 (HLA-A*01:01, HLA-A*01:02, HLA-A*25:01, HLA-A*30:04, HLA-A*32:01, HLA-A*36:01, HLA-A*43:01, HLA-A*80:01), HLA-A*02 (HLA-A*02:01, HLA-A*02:02, HLA-A*02:03, HLA-A*02:04, HLA-A*02:05, HLA-A*02:06, HLA-A*02:07, HLA-A*02:09, HLA-A*02:14, HLA-A*02:17, HLA-A*68:02, HLA-A*69:01), HLA-A*03 (HLA-A*03:01, HLA-A*11:01, HLA-A*31:01, HLA-A*33:01, HLA-A*33:03, HLA-A*34:02, HLA-A*66:01, HLA-A*68:01), HLA-A*24 (HLA-A*24:02, HLA-A*24:03, HLA-A*24:04, HLA-A*23:01, HLA-A*30:01, HLA-A*30:02, HLA-A*30:03), HLA-A*26 (HLA-A*26:01, HLA-A*26:02, HLA-A*26:03, HLA-A*26:04), and HLA-B*07 (HLA-B*07:02, HLA-B*07:03, HLA-B*07:04, HLA-B*07:05, HLA-B*15:08, HLA-B*35:01, HLA-B*35:02, HLA-B*35:03, HLA-B*51:01, HLA-B*51:02, HLA-B*53:01, HLA-B*54:01, HLA-B*55:01, HLA-B*55:02, HLA-B*56:01, HLA-B*56:02, HLA-B*67:01, HLA-B*78:01) (18, 19).

### 2.2 Peptide microarray for the assessment of HLA-A*02-restricted peptide signature *in vitro*

To assess the HLA-A*02-restricted peptide signature, a customized microarray was performed by PEPperPRINT^©^ (Heidelberg, Germany) as described previously (20) with modifications. Briefly, 2,555 peptides from HCoV-NL63, MERS-CoV, SARS-CoV-1, and SARS-CoV-2 were printed on a PEPperCHIP^©^ microarray slide and incubated with a HLA-A*02:01 dimeric protein (Ig : DimerX - BD Biosciences, California, USA) at 1µg/mL or 10µg/mL diluted in staining buffer (PBS + 10% bovine serum albumin). After 16 hours, the slide was washed and incubated with anti-murine IgG1/Cy3 secondary antibody (BD Biosciences, San Jose, CA, USA) diluted in staining buffer (1:5,000). After 45 minutes of incubation, the secondary antibody was removed. The slide was air-dried and digitized using the Affymetrix 428 Array Scanner device (Thermo Fisher, California, USA). Peptide reactivity to HLA-A*02 was analyzed using PepSlide^®^ Analyzer (PEPperPRINT ^©^, Heidelberg, Germany). Quantification was performed using 16-bit grey-scale images and results were expressed in fluorescence intensity.

### 2.3 Prediction of cytokine induction and physicochemical parameters by HLA-A2-restricted peptides

HLA-A2-restricted peptides derived from HCoV-NL63, MERS-CoV, SARS-CoV-1, and SARS-CoV-2 with Combined Score (CS) ≥0.75 were examined for IFN-γ and IL-4 induction in silico by MHC-II axis with IFN epitope (https://webs.iiitd.edu.in/raghava/ifnepitope/index.php) and IL4pred (https://webs.iiitd.edu.in/raghava/il4pred/index.php) software, applying Support Vector Machine method (21, 22). Peptides were also assessed for physicochemical parameters in IL4pred (https://webs.iiitd.edu.in/raghava/il4pred/index.php) (21).

### 2.4 Data mining and statistical analysis

Peptides with HLA-A2 reactivity *in vitro* that were shared between two or more species of coronaviruses included in this study were identified using Venn diagram (http://bioinformatics.psb.ugent.be/webtools/Venn/).

Logo sequence analysis was constructed using WebLogo 3 (https://weblogo.threeplusone.com/) to assess conserved residues in HLA-A*02:01 reactive peptides that are either lost or gained in Alpha, Beta, Delta, Gamma, and Omicron SARS-CoV-2 variants (VOCs) when compared to the reference Wuhan sequence (23).

Correlations between the peptide potential of IFN-γ or IL-4 induction and physicochemical properties were assessed by Spearman’s rank correlation test on GraphPad Prism v.8.0 software (GraphPad Software, California, USA). Correlation networks were assembled using Cytoscape v3.9.0 software (24).

Orange3-3.34.0 software was used for the generation of tSNE and heatmap analysis of peptide potential of IFN-γ and IL-4 induction and physicochemical properties using normalized values (25).

Additional graphical and statistical analyses were created using GraphPad Prism v.8.0 software (GraphPad Software, California, USA).

Non-parametric distribution of the dataset was confirmed by the Shapiro-Wilk test, whereas differences between groups were assessed by Mann-Whitney, Wilcoxon, or Kruskal-Wallis followed by Dunn’s post-test. Statistical significance was considered at p<0.05 and is indicated by asterisks in the graphs.

## 3 Results

### 3.1 Predominance of immunodominant peptides in human coronaviruses: highest MHC binding profile exhibited by SARS-CoV-2 Wuhan strain

**Figure 2:**
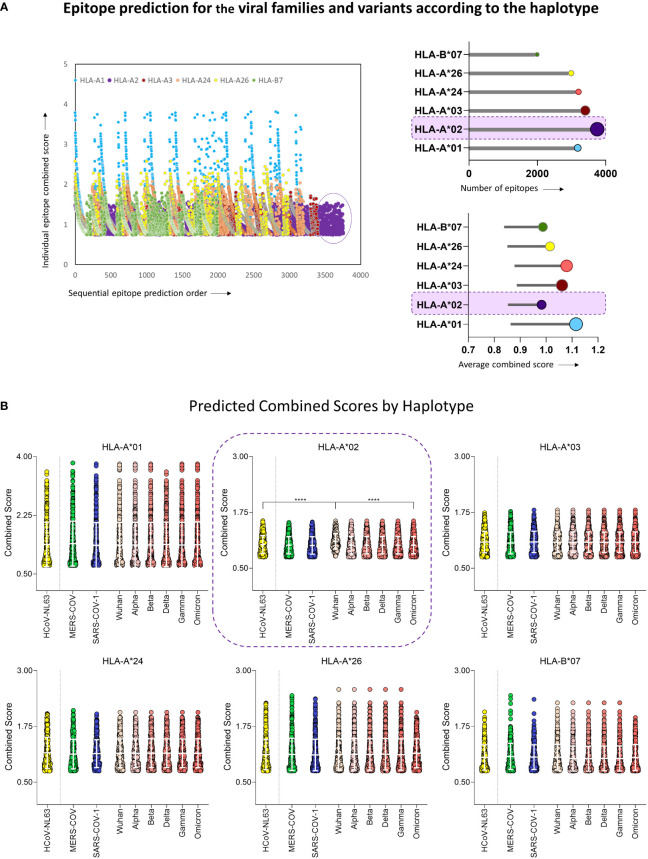
Epitope Prediction for the Viral Families and Variants According to the Haplotype. **(A)** Combined prediction score value for all predicted epitopes above 0.75 (Left panel). Lollipop graphs with total number of predicted peptides (upper right panel) and the average value of peptide combined prediction score (lower right panel) for all six MHC-I haplotypes included in this study, namely HLA-A*01, HLA-A*02, HLA-A*03, HLA-A*24, HLA-A*26, HLA-B*07. The Haplotype with the highest number of predicted peptides is highlighted in both graphs. **(B)** Violin plot with individual values of predicted combined score for peptides derived from HCoV-NL63, MERS-CoV, SARS-CoV-1, and six variants of SARS-CoV-2 (Wuhan reference sequence, Alpha, Beta, Delta, Gamma, and Omicron) for haplotypes HLA-A*01, HLA-A*02, HLA-A*03, HLA-A*24, HLA-A*26, HLA-B*07. White bars indicate mean with standard deviation. Multiple comparisons among groups were analyzed by Kruskal-Wallis test followed by Dunn’s post-test. Statistical significance was considered for p<0.05 and is indicated by bars and asterisks (**** = p<0.0001).

To assess the ability of human coronaviruses (HCoV) and their viral peptides to being immunogenic, a prediction of peptide and human MHC binding was performed using protein sequences from HCoV-NL63, MERS-CoV, SARS-CoV-1 and six strains of SARS-CoV-2 (Wuhan reference sequence, and VOCs Alpha, Beta, Delta, Gamma, and Omicron). The results in **Figure 2** show the ability of these peptides to bind with restriction to the six most prevalent MHC-I worldwide (HLA-A*01, HLA-A*02, HLA-A*03, HLA-A*24, HLA-A*26, and HLA-B*07). The results demonstrate a predominance of HLA-A*02-restricted peptides in HCoV (**Figures 2A, B** upper panel), which indicates that this haplotype responds majorly to coronaviruses. However, it was also possible to observe that HLA-A*02-restricted peptides presented the lowest values of combined prediction score, with a median of 0.976 (**Figure 2B** lower panel), in comparison with the results obtained for other MHC-I.

Furthermore, Wuhan original strain showed the highest binding scores when compared to other variants (**Figure 2B**). Considering these results and the predominance of HLA-A*02 in the population, peptides restricted to this haplotype were selected for further scrutiny (18, 26). These results clearly indicate that, while mutations occur along time, variants are probably losing immunodominant regions within the virus genome as compared to the original strain, suggesting loss of viral immunogenicity along time.

### 3.2 HLA-A*02-restricted SARS-CoV-2 peptide signature indicates poor overlap of reactive peptides amongst human coronaviruses

**Figure 3:**
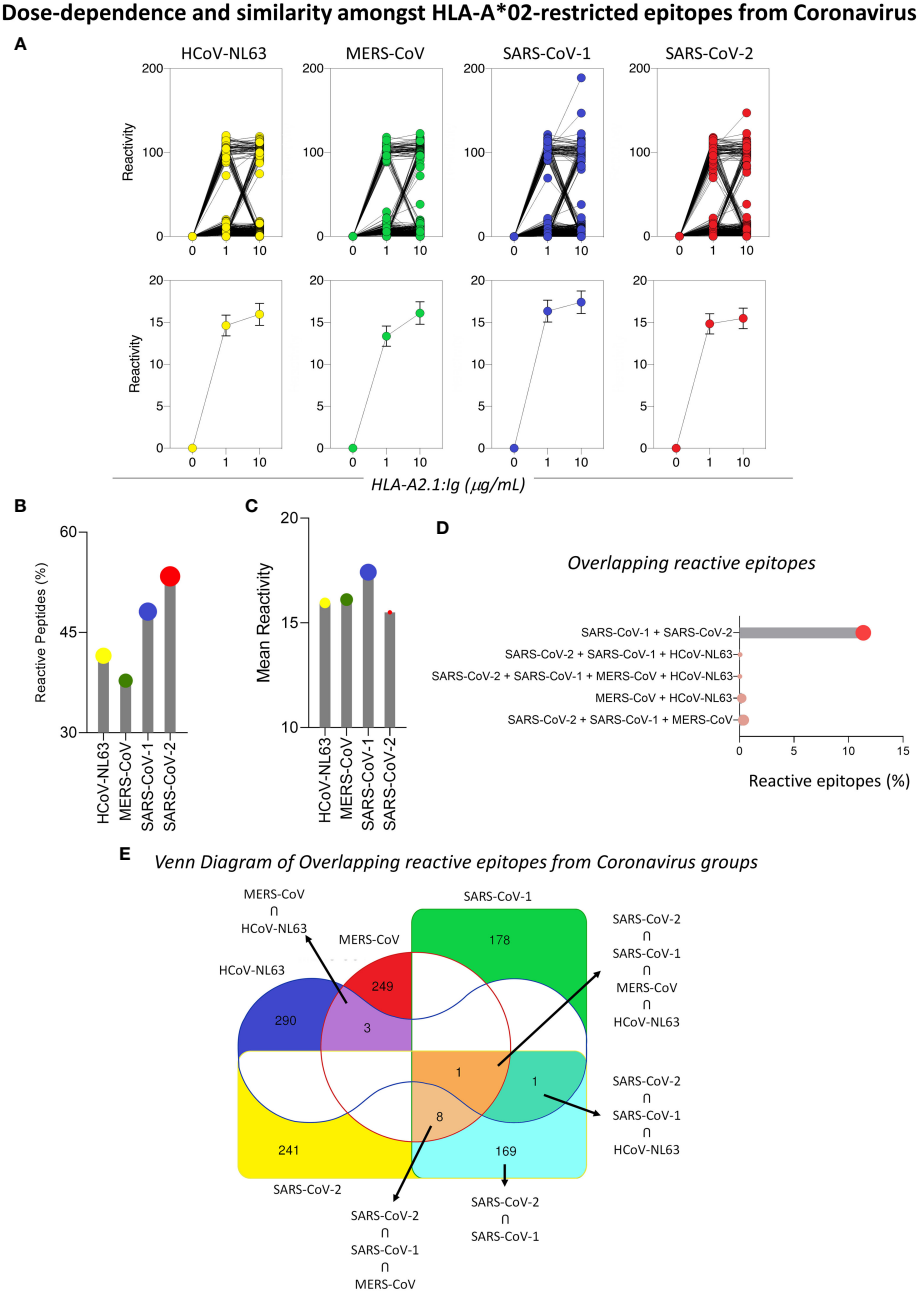
Dose-Dependence and Similarity Amongst HLA-A*02-restricted Epitopes from Coronavirus. **(A)** *In vitro* reactivity of HLA-A*02-restricted peptides derived from HCoV-NL63, MERS-CoV, SARS-CoV-1, and SARS-CoV-2 in the peptide microarray for two HLA-A*02:01:Ig concentrations (1μg/mL and 10μg/mL), expressed as individual values (upper panel) and mean with standard deviation (lower panel). **(B)** Lollipop graphs for the percentage of HLA-A2*01-restricted reactive peptides and mean of peptide reactivity in different species of coronaviruses (HCoV-NL63, MERS-CoV, SARS-CoV-1 and SARS-CoV-2) for 10ug/mL **(C)**. Percentage of HLA-A*02 reactive peptides in the peptide microarray that are shared between two or more species of coronaviruses included in this study **(D)**. **(E)** Venn diagram with total number of reactive HLA-A*02 peptides derived from HCoV-NL63, MERS-CoV, SARS-CoV-1, and SARS-CoV-2 and shared between two or more species of coronaviruses. Regions of peptide overlapping are highlighted by colors (red – MERS-CoV; green – SARS-CoV-1; blue – HCoV-NL63; yellow – SARS-CoV-2).

In order to confirm the in silico results for the prediction of loss in viral immunogenicity, a analysis of peptide signatures was carried out by the analysis of peptide-HLA-A*02 reactivity on a microarray chip containing selected SARS-CoV-2 peptide sequences. The results showed a dose-dependence binding for the majority of the peptides tested, with higher means of reactivity at 10µg/mL of HLA-A*02:Ig than at 1µg/mL (**Figure 3A** upper panels), which corroborates the in silico results. Moreover, a comprehensive analysis of the HLA-A*02:Ig-reactive peptides in all four coronavirus species revealed abundant reactive peptides, with lower percentages for SARS-CoV-1 and SARS-CoV-2 in comparison to HCoV-NL63 and MERS-CoV (**Figure 3** left panel). However, despite the low percentage of reactive peptides, SARS-CoV-1 presented the highest mean of *in vitro* HLA-A*02:Ig reactivity, whereas SARS-CoV-2 presented the lowest values of overall reactivity (**Figure 3** right panel), indicating possible HLA-A*02 reactive peptide impairment in inducing CD8^+^ T cell responses as compared to other coronaviruses.

Considering the phylogenetic proximity of coronaviruses studied, we searched for possible overlapping reactive peptides amongst two or more species of coronaviruses. To obtain a panoramic view of overlapping HLA-A*02-reactive peptides, a Venn diagram was built using in silico tools. Colored regions specify the occurrence of shared reactive peptides between HCoV, whereas white regions mark viruses for which there was no overlapping reactive peptides. Interestingly, the results showed that a minority of reactive peptides presented full overlapping sequences for two or more coronaviruses, as shown in **Figure 3E**. The highest rate of overlap was observed between SARS-CoV-1 and SARS-CoV-2 (11.01%) (**Figures 3D, E**).

### 3.3 A predominance of HLA-A*02:Ig-reactive peptides of SARS-CoV-2 from non-structural proteins and from the nucleocapsid protein

**Figure 4:**
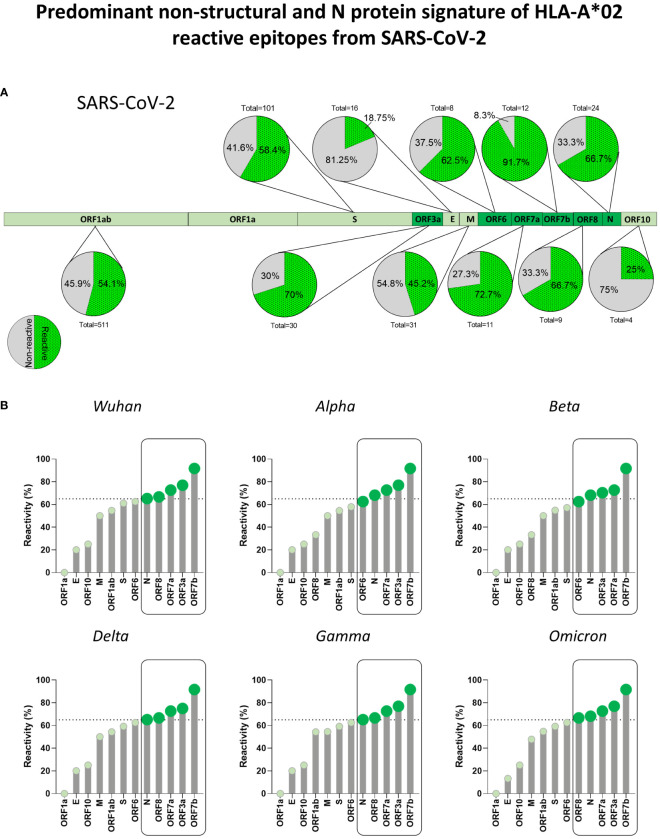
Predominant Non-Structural and N Protein Signature of HLA-A*02 Reactive Epitopes from SARS-CoV-2. **(A)** Percentage of HLA-A*02:Ig reactive peptides derived from structural and non-structural proteins of SARS-CoV-2, as indicated by pie charts. Green dotted region of pie charts indicates reactive peptides and grey regions indicate non-reactive peptides. Total number of peptides tested for *in vitro* reactivity is also indicated for every protein. Regions with the highest percentage of reactive peptides are highlighted by dark green in the protein schematics. **(B)** Percentage of reactive HLA-A*02:Ig reactive peptides divided by viral protein in the Wuhan reference sequence as well as for Alpha, Beta, Gamma, Delta, and Omicron variants. Dotted lines in lollipop graphs indicate high percentage of reactive peptides (upper tercile). Protein regions with a percentage of reactive peptides above that threshold are indicated by dark green and grouped by black rectangles in all assessed SARS-CoV-2 variants.

To determine which SARS-CoV-2 viral proteins display immunodominant peptides, epitope mapping of HLA-A*02:Ig reactive peptides was assessed for every viral protein. **Figure 4A** brings a representative scheme of all SARS-CoV-2 proteins and pie charts display the total number of peptides as well as the percentage of reactive peptides. Considering all assessed SARS-CoV-2 variants, a higher percentage of reactive peptides was observed for ORF7b (91.7%), ORF7a (72.2%), ORF3a (70%), ORF 8 (66.7%), N (66.7%) and ORF6 (62.5%), followed by S protein (58.4%), as shown in **Figure 4A**. Dark green regions highlight proteins with more than 60% of reactive peptides.

However, whereas ORF7b, ORF3a, ORF7a, ORF8, and N proteins were the top 5 main sites of HLA-A*02:Ig reactive peptides for the Wuhan, Delta and Gamma variants, the Omicron variant showed a slight pattern shift, with N protein representing main hotspot as shown in lollipop graphs (**Figure 4B**).

### 3.4 ORF8 and spike protein are major sites of HLA-A*02:Ig-reactive peptides loss in SARS-COV-2 variants in comparison to the Wuhan original strain

**Figure 5:**
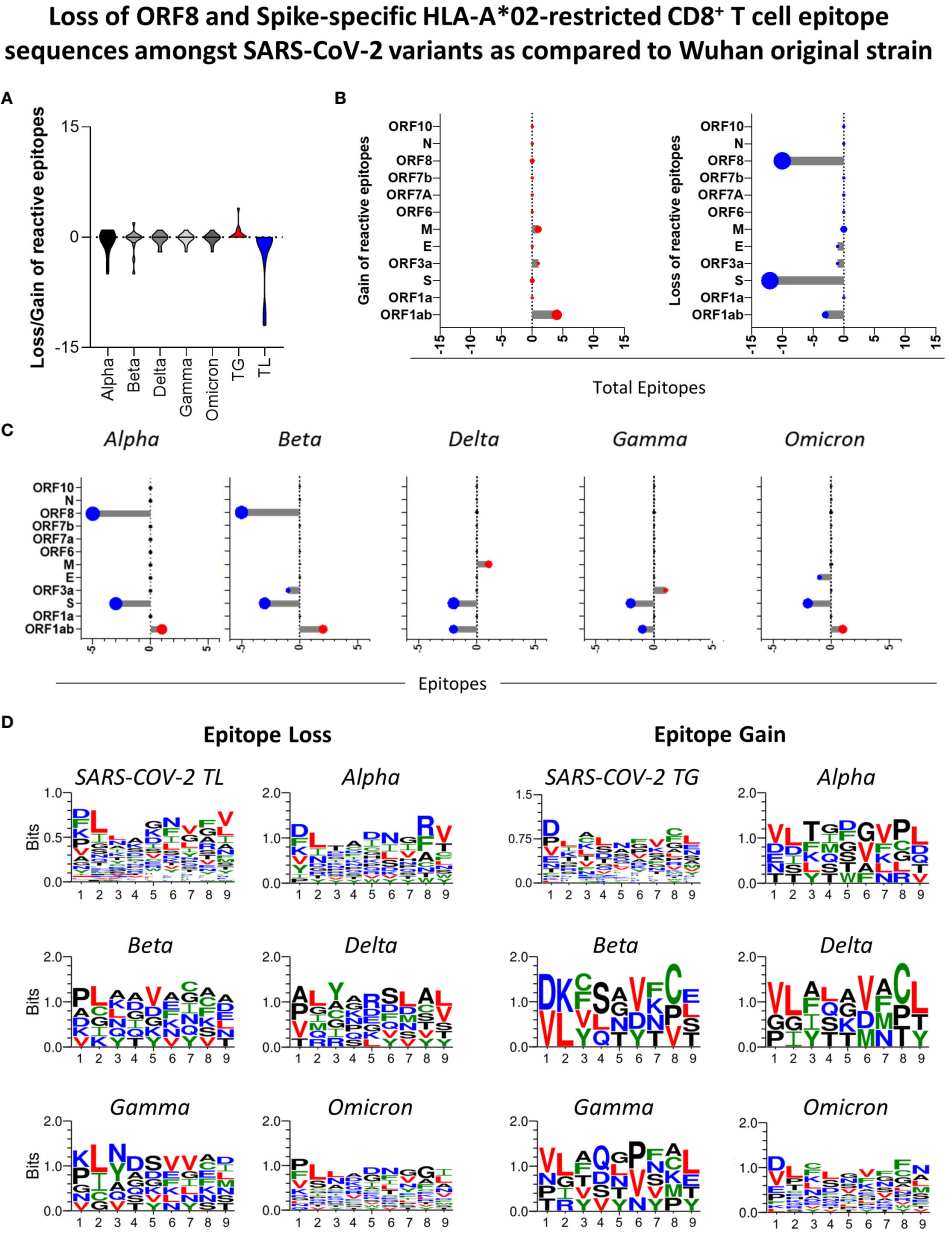
Loss of ORF8 and Spike-Specific HLA-A*02-restricted CD8^+^T cell Epitope Sequences amongst SARS-CoV-2 Variants as Compared to Wuhan Original Strain. **(A)** Number of HLA-A*02:Ig reactive epitope loss or gain in variants Alpha, Beta, Delta, Gamma, and Omicron in comparison to Wuhan original strain, as well as total gain (TG) and total loss (TL) of HLA-A*02:Ig reactive epitopes in all assessed variants. **(B)** Number of gained (left panel) or lost (right panel) reactive peptides in all viral proteins for Alpha, Beta, Delta, Gamma, and Omicron variants taken together and separately **(C)**. **(D)** Logo sequence analysis for the evaluation of conserved residues in peptides that were either lost (left panel) or gained (right panel) in all assessed variants taken together (SARS-CoV-2 TL or SARS-CoV-2 TG, respectively) and in Alpha, Beta, Delta, Gamma and Omicron variants separately in comparison to Wuhan original strain. The size of the amino acid abbreviation is related to its overall presence in the gained/lost peptides. X-axis indicates the amino acid position within peptide sequence. Hydrophilic residues are indicated in blue, hydrophobic residues in green, neutral residues in black, and leucine (L) and valine (V) are highlighted in red.

Panoramic assessment of HLA-A*02:Ig-reactive peptide signatures for SARS-CoV-2 VOCs (Alpha, Beta, Delta, Gamma and Omicron) revealed the occurrence of peptide gain and loss, in comparison to the Wuhan original strain. Peptide gain was defined as reactive peptides that are encoded in VOCs strains but were not identified in the Wuhan original strain. Conversely, peptide loss was identified when a specific reactive peptide was derived from the Wuhan sequence but was not found amongst the VOCs. Overall, peptide loss was greater than peptide gain for the 5 variants taken together (**Figure 5A**). The major sites in which such losses occurred were identified as ORF8 and S protein for all assessed VOCs (**Figure 5B** right panel). However, peptide loss was observed mainly for S protein in Delta, Gamma, and Omicron variants (**Figure 5C**). Peptide gain was less common and occurred mostly in ORF1ab, but also in ORF3 and M protein (**Figure 5B** left panel and 5C).

Next, we aimed at assessing distinct amino acid patterns in peptides that were either lost or gained in SARS-CoV-2 VOCs using logo sequence analysis. For this analysis, the size of the amino acid symbols is directly correlated to the frequency of occurrence of each residue in specific positions within the peptide sequence. Hydrophilic residues are indicated in blue, hydrophobic residues in green, neutral residues in black, and leucine (L) and valine (V) are highlighted in red. In this case, the totality of lost (TL) and gained (TG) peptides showed a similar pattern on conserved amino acid residues, but it was possible to distinguish a conspicuous loss of peptides with leucine (L) in position 2, especially for Beta and Gamma (**Figure 5D** right panel). For all SARS-CoV-2 variants evaluated, gained peptides (TG) display aspartic acid (D) as a predominant residue in the peptide sequence (**Figure 5D** left panel). As expected, the Omicron variant presented the highest shuffling of peptide residues in all 9 positions, both for gained and lost peptides (**Figure 5D**). These results indicate that mutations in VOCs are causing non silent amino acid sequence changes on CD8 T cell epitopes, which elucidate and reflect on the VOC immunogenicity loss.

### 3.5 HLA-A*02-restricted peptides derived from human coronaviruses tend to induce Th2 as opposed to Th1 profile

**Figure 6:**
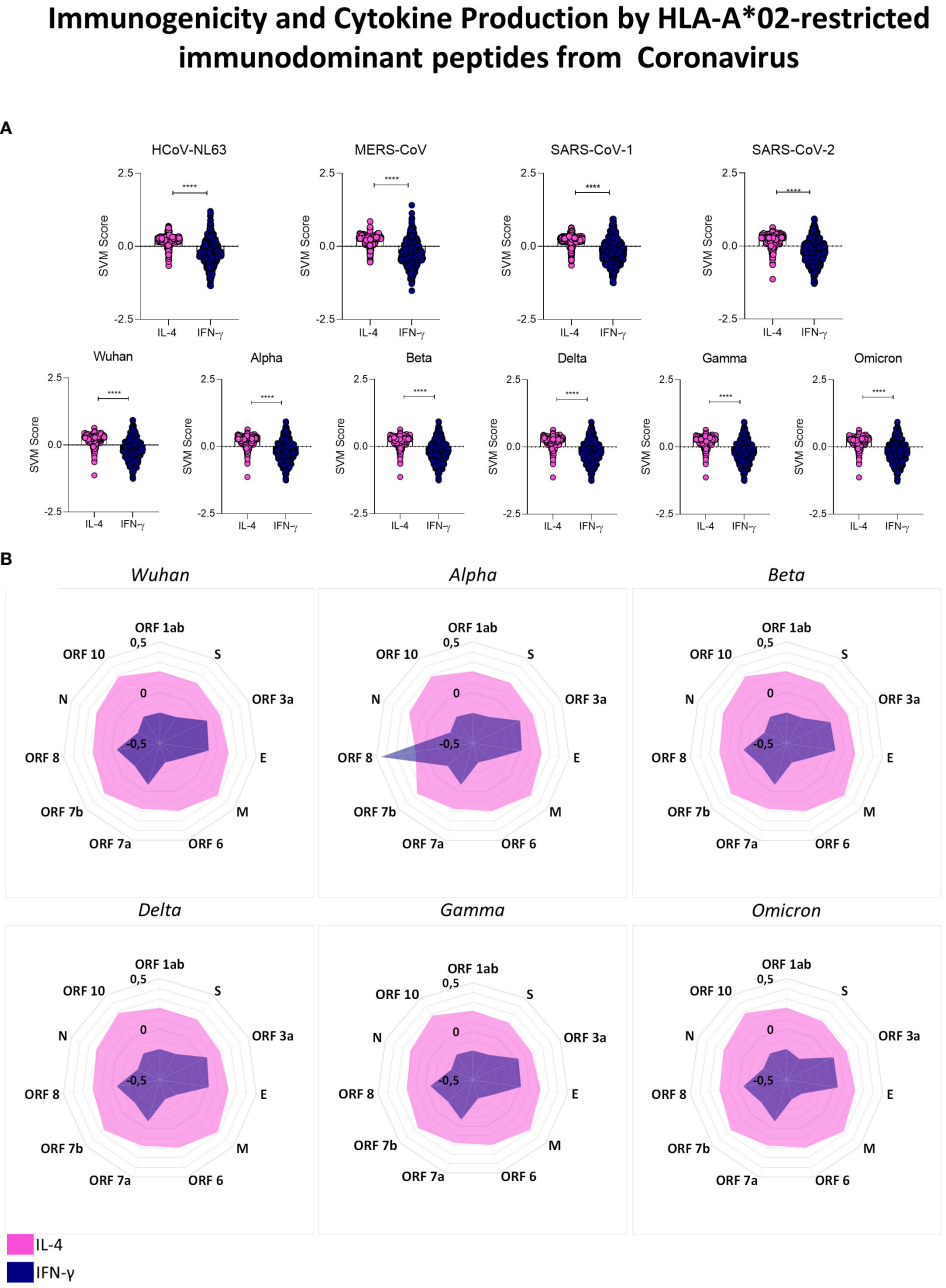
Immunogenicity and Cytokine Production by HLA-A*02-restricted Immunodominant Peptides from Coronavirus. **(A)** Overall predicted score of IL-4 (pink) or IFN-γ (blue) induction by HLA-A*02-restricted peptides in HCoV-NL63, MERS-CoV, SARS-CoV-1 and SARS-CoV-2 (upper panel) and individually in SARS-CoV-2 Wuhan original strain and in Alpha, Beta, Delta Gamma and Omicron variants (lower panels). Comparison between groups was assessed by Wilcoxon. Statistical significances were considered for p>0.05 and are indicated by bars and asterisks (**** = p<0.0001). **(B)** Radar charts showing the mean of the predicted score for IL-4 (pink region) or IFN-γ (blue region) induction by HLA-A*02-restricted peptides derived from all viral proteins in SARS-CoV-2 Wuhan original strain and in Alpha, Beta, Delta, Gamma and Omicron variants.

Aiming at understanding the implications of HLA-A*02-restricted peptide presentation, we performed an in silico assessment of cytokine induction by these peptides, focusing on the induction of Th1 (IFN-γ) and Th2 (IL-4) mediators. Our results highlighted a dominant tendency of reactive peptides to induce IL-4 (pink) in comparison to IFN-γ (blue) with robust statistical significance for HLA-A*02-restricted peptides derived from all evaluated coronavirus included in the study (**Figure 6A**) (p<0.0001 = ****). The assessment of cytokine induction by HLA-A*02-restricted peptides derived from different proteins of SARS-CoV-2 was displayed in radar charts in which the axis indicates all structural and non-structural viral proteins and the extent of the spokes indicate the score of cytokine production (SVM score). The observed results confirmed the predominance of IL-4 induction by structural and non-structural proteins of Wuhan original strain as well as for all five SARS-CoV-2 variants (**Figure 6B**). The only exception was detected for ORF8 from the Alpha variant, with a strikingly higher potential for IFN-γ induction (**Figure 6B**).

### 3.6 Overall assessment of predicted physicochemical parameters revealed clusters of hydrophobic peptides with increased potential of IFN-γ induction

**Figure 7:**
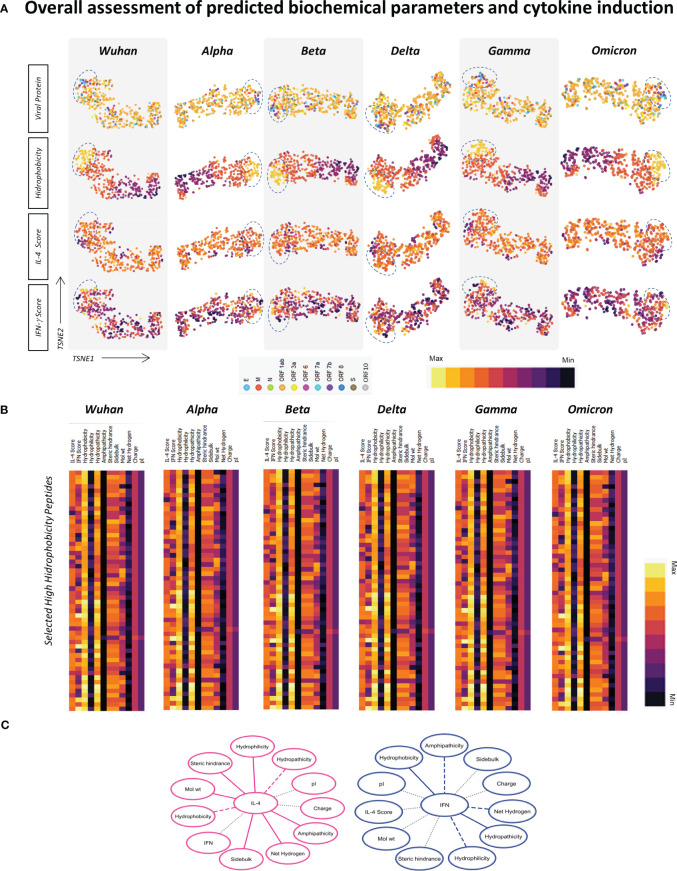
Overall Assessment of Predicted Biochemical Parameters and Cytokine Induction. **(A)** t-SNE analysis of SARS-CoV-2 Wuhan original strain and Alpha, Beta, Delta, Gamma, and Omicron variants, displaying the location of HLA-A*02-restricted peptide (viral protein) and normalized scores of hydrophobicity, prediction of IL-4 induction and prediction of IFN-γ induction. A cluster of peptides with high hydrophobicity is highlighted by dotted lines. **(B)** Heatmap analysis of normalized physicochemical parameters such as hydrophobicity, hydrophilicity, hydrophaticity, amphipaticity, steric hindrance, side bulk, molecular weight, net hydrogen, charge, and isoelectric point (pI) as well as predicted scores for IL-4 and IFN-γ induction in clustered peptides of high hydrophobicity for SARS-COV-2 Wuhan original strain and Alpha, Beta, Delta, Gamma, and Omicron variants. **(C)** Network correlations between in silico scores of cytokine induction (IL-4 or IFN-γ) and physicochemical properties of HLA-A*02-restricted peptides. Complete lines represent positive correlations with statistical significance (p<0.05), and dash lines represent negative correlations with statistical significance (p<0.05). Dotted grey lines represent correlations with no statistical significance (p>0.05).

Considering that parameters such as hydrophobicity, hydrophilicity, isoelectric point, and molecular weight can affect the potential of an epitope to elicit different shades of the immune response, the next step was to apply tSNE analysis for the assessment of physicochemical properties of HLA-A*02-restricted peptides derive from SARS-CoV-2 (Wuhan reference sequence and assessed VOCs, Alpha, Beta, Delta, Gamma and Omicron) regarding their potential to induce IL-4 and/or IFN-γ. In this strategy, every dot represents a peptide, and the color pattern is associated with the in silico score obtained for each assessed parameter in each of the tSNE graphs. In order to grant a simultaneous overview of all physicochemical parameters in all VOCs and for the Wuhan reference virus, heat map graphs were also assembled. This strategy allowed the identification of a cluster of hydrophobic peptides with increased potential of IFN-γ induction in comparison to other peptides (**Figures 7A, B**). These peptides also presented medium/low potential for IL-4 induction (**Figures 6A, B**) and were derived both from structural and non-structural proteins from all SARS-CoV-2 variants included in this study and the Wuhan original strain (**Figure 6A**).

Furthermore, correlation networks based on r Spearman’s test showed a positive correlation (full lines) between peptide induction of IFN-γ and hydrophobicity and a negative correlation (dotted lines) between IFN-γ induction and peptide hydrophilicity, whereas the opposite was observed for IL-4 induction (**Figure 7C**). These results may bring some light on the design of vaccine strategies for coronaviruses, considering the inclusion of viral peptides able of inducing IFN-γ, which may be lost by new VOCs and are essential for promoting a protective and long-lasting anti-SARS-CoV-2 immune response.

### 3.7 Evaluation of peptide signature of Omicron subvariants

**Figure 8:**
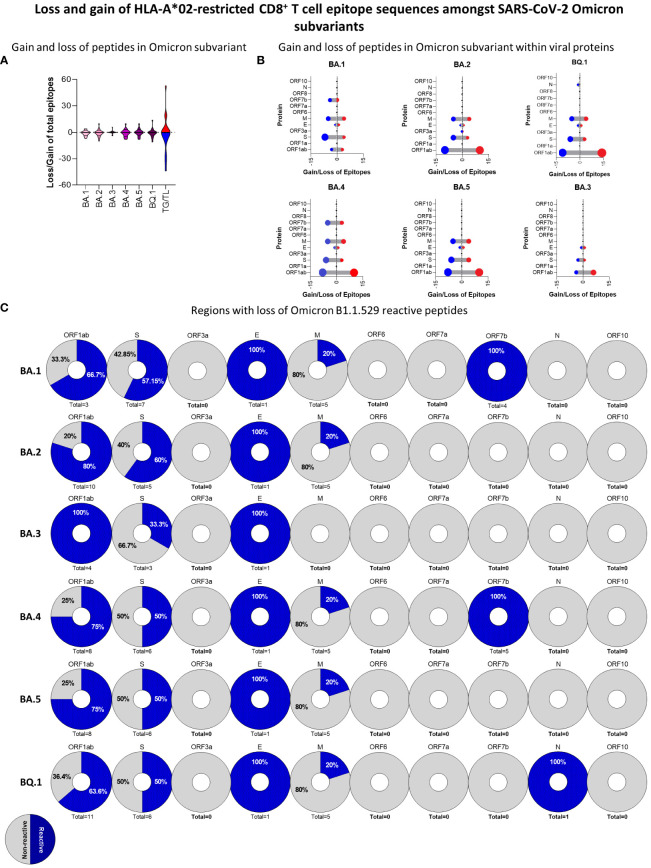
Loss and Gain of HLA-A*02-Restricted CD8^+^ T-Cell Epitope Sequences Amongst SARS-CoV-2 Omicron Subvariants. **(A)** Overall number of gained and lost HLA-A*02-restricted peptides for BA.1, BA.2, BA.3, BA.4 BA.5, and BQ.1 Omicron subvariants in comparison to Wuhan original strains. Number of total gain (TG) and total loss (TL) of peptides is also indicated in violin plots. **(B)** Lollipop graph indicating the number of gained (red) or lost (blue) HLA-A*02-restricted peptides in every viral protein for BA.1, BA.2, BA.3, BA.4, BA.5, and BQ.1 Omicron substrains. **(C)** Percentage of loss HLA-A*02:Ig reactive peptides in BA.1, BA.2, BA.3, BA.4 BA.5, and BQ.1 Omicron subvariants in comparison to Wuhan original strains. Every pie chart indicates one viral protein containing the total number of loss peptides. Blue dotted regions indicate the percentage of HLA-A*02:Ig reactive peptides, grey indicates the percentage of non-reactive peptides per region.

Omicron or B1.1.529 variant has become dominant as SARS-CoV-2 continues to spread worldwide. This could be attributed to the emergence of subvariants of concern with superior viral fitness and transmissibility as well as evasion from previous immune mechanisms induced by either disease or vaccination. Therefore, it is essential to address this by performing a detailed analysis of the HLA-A*02-restricted peptide signature of the new Omicron subvariants. For that, we carried out peptide prediction for six new Omicron subvariants (BA.1, BA.2, BA.3, BA.4, BA.5 and BQ.1) and assessed the rate of peptide gain and loss in comparison to the original Wuhan strain (**Figure 8A**). Overall, all the subvariants presented significant changes as compared to the original strain, apart from BA.3, which remained most similar to the Wuhan strain (**Figure 8A**). A more detailed viral protein mapping of gained and lost peptides revealed ORF1ab as a major site of peptide changes on BA.1, BA.2, BA.4, BA.5, and BQ.1 subvariants in comparison to the Wuhan strain (**Figure 8B**). Meanwhile, all Omicron subvariants had immunodominant peptide loss rather than gain on S protein, but especially for BA.1 (n=7), BA.4 (n=6) BA.5 (n=6), and BQ.1 (n=6) (**Figure 8B**).

Next, we assessed the percentage of peptides that presented *in vitro* reactivity to the HLA-A*02:Ig molecule and were derived from the Wuhan reference sequence but were lost on Omicron subvariants. The results are shown as pie charts assembled with the total number of lost peptides derived from each SARS-CoV-2 protein from Omicron subvariants in comparison to the Wuhan reference sequence. Data analysis showed that most peptides lost in the ORF1ab region were reactive in the *in vitro* assay. Interestingly, 20% of lost peptides were derived from the M protein (**Figure 8C**). As for the S protein, the first subvariants, BA.1 and BA.2, have lost more HLA-A*02:Ig-reactive peptides in this protein sequence, while BA.3 lost only one HLA-A*02:Ig-reactive peptide in this region (**Figure 8C**). These results indicate that the loss of immunodominant epitopes may follow a specific dynamics in which changes in S protein immunodominance are observed early on during viral divergence.

## 4 Discussion

The development of robust CD8^+^ T cell responses during COVID-19 is already reported as a mechanism of protection against viral spread within the host and viral clearance may be dependent upon the cytolytic effect of these cells for eliminating viral reservoirs together with anti-SARS-CoV-2 antibody and CD4 T cell responses (8–11). Thus, it is essential to consider CD8^+^ T cell subsets in inducing duration of protective memory responses against SARS-CoV-2. Therefore, the present work combined in silico and *in vitro* approaches to provide a snapshot of the CD8^+^ T cell epitope mapping of SARS-CoV-2 peptide signatures. To broaden the present investigation, HCoV-NL63, MERS-CoV, and SARS-CoV-1 were also included. For the SARS-CoV-2 peptides signature analysis, Wuhan original strain was addressed as a reference for comparison with the six most prevalent variants of concern (VOCs) (Alpha, Beta, Delta, Gamma, and Omicron) (27).

Peptide prediction showed that even with a clear bias towards HLA-A*02-restricted peptides, these sequences presented lower binding score when compared to other MHC-I haplotypes with high prevalence worldwide (18, 26, 28) (**Figure 2A**). Previous reports have shown that peptide-HLA-A*02 affinity could be associated with a direct impairment of CD8^+^ T-cell activation (29, 30). In this case, the requirement for additional stimulatory signals triggered by CD4^+^ T cells or APCs would be required for low-affinity peptides (31) but could be hampered by the lymphopenia and dendritic cell deficiency observed in patients with COVID-19 (32, 33). Furthermore, all five SARS-CoV-2 VOCs presented lower CS for HLA-A*02-restricted peptides than the Wuhan reference sequence, which could be important for the increase of disease severity observed for Alpha, Beta, Delta, and Gamma variants as well as for the elevated transmission associated with Omicron worldwide (5, 34–37) (**Figure 2B**).

Despite this observation, and previous works indicate a suboptimal CD8^+^ T cell response against SARS-CoV-2 in HLA-A2^+^ individuals since the beginning of COVID-19 pandemic (30). To our knowledge, there is no well-founded correlation between HLA-A*02 and SARS-CoV-2 disease severity (38). In fact, data regarding MHC-I/MHC-II haplotypes and SARS-CoV-2 disease outcome is still contradictory and the observed correlations may vary between distinct populations with different frequencies of HLA haplotype compositions (38–41).

In agreement to our findings, previous reports of SARS-CoV-2 mutations that alter the presentation of viral peptides by HLA molecules found that a essential number of peptides tightly bound to HLA-B*07:02 in the Wuhan variant ceased to be tight binders for the Indian (Delta) and the UK (Alpha) variants, suggesting loss of CD8^+^ T cell epitope dominance and affinity (16). Nonetheless, it is important to highlight that although robust in silico methods were used to map SARS-CoV-2 T-cell epitopes, *in vitro* validation is still required. Our study brings additional and reliable *in vitro* methods to confirm HLA-A2 MHC-I binding, which adds information to the T-cell epitope loss during SARS-CoV-2 mutation and evolution. Therefore, we believe that the novelty in our findings is based on the combination of in silico and *in vitro* methods to confirm CD8^+^ T cell epitope loss in SARS-CoV-2 variants. We believe that these results should be taken together with B cell epitope mapping to bring awareness to the loss of SARS-CoV-2 immunogenicity as well as to improve vaccine design for future COVID immunoprophylactic strategies. Expanding the techniques utilized here for other haplotypes is crucial for tracing a wider map of SARS-CoV-2 immunogenicity variations along the viral variants evolution.

Regarding the cross-reactivity of CD4^+^ T cells and CD8^+^ T cells previously described for SARS-CoV-2 and other coronaviruses (42), we found little overlap of reactive HLA-A*02-restricted peptides for the HCoV evaluated in this work, with most shared peptides identified between SARS-CoV-2 and SARS-CoV-1(**Figures 3D, E**). This result demonstrates the specificity of the antiviral CD8^+^ T cell response incited against SARS-CoV-2, indicating that cross-reactivity, if existent, may be restricted and grant protection of HLA-A*02^+^ individuals against severe cases of COVID-19 but not against SARS-CoV-2 infection (42, 43).

Another interesting aspect of the HLA-A*02-peptide signature was the predilection of this haplotype for peptides derived from non-structural proteins 1-16 in ORF1ab (n = 511) (29). However, the ratio of ORF1ab-derived peptides that were capable to bind to HLA-A*02 in the *in vitro* assay was approximately 50% for all VOCs, whereas N protein and ORFs 3a, 6, 7a, 7b, 8 presented reactivity of 60% or more (upper tercile) (**Figure 4B**) Previous reports have shown that ORFs 3a, 6, 7a, and 8 are important immunomodulatory regions, capable of inducing MHC-I downregulation by different pathways and decreasing CTL response directed at these proteins (44–46). Therefore, this data suggests that in addition to the S protein, N, and ORF7b, a conserved accessory protein (47) could be promising targets for the development of CD8^+^ T-cell response. Moreover, N protein was already described as a conserved and abundant protein during SARS-CoV-2 infection (48), and vaccines aimed at this target have already started to be tested in animal models with relative success (49, 50).

ORF1ab comprises almost 1/3 of the virus proteome, accumulating important mutations (51–53), that is most likely centered in specific non-structural proteins since many conserved sites were identified (52). In this regard, it was somewhat expected that CD8^+^ T cell peptide gain could occur in this region. Even so, peptide gain was extremely limited, and the loss of reactive peptides surpassed the number of gained ones in Omicron subvariants (**Figure 8**).

The rates for S-derived peptide reactivity remained below 60% for SARS-CoV-2. S protein was another major site of CD8^+^ T-cell epitope loss for the assessed VOCs in comparison to the Wuhan reference strain (**Figures 5B, C**). All VOCs included in this study have emerged from mutations in the S protein of the Wuhan strain (35) that had already been described as means of immune evasion for neutralizing antibodies (54, 55), but this data indicates that the antiviral cellular immune response against S protein could also be compromised, explaining the protection against severe cases of COVID-19, but not against viral infection (56). Such prospect associated with the rapid emergence of new SARS-CoV-2 variants is worrisome in the context of mass immunization with vaccines targeting S protein, particularly in developing countries, where updated vaccines can take longer to be available and new variants may spread quicker. Furthermore, the constant need for vaccine boosts presents a worldwide challenge to retain the engagement of the population, which can lead to insufficient vaccinal coverage against the new variants even in developed countries. All this taken together suggests that S-based vaccines may not be time or cost-effective anymore and new immunization targets are needed.

A detailed evaluation of acquired and lost HLA-A*02:Ig-reactive peptides in the five SARS-CoV-2 VOCs showed ORF8 as a “hot-spot” for CD8^+^ T-cell epitope loss, especially in Alpha and Beta variants (**Figure 5C**). These results corroborate previous reports that show ORF8 as the most variable accessory protein of SARS-CoV-2, with several of its mutations correlated to mild cases of COVID-19 (57). Likewise, Alpha and Beta variants were two of the most similar variants regarding clinical outcomes and presented lower disease severity in comparison to Delta variants in some countries (58).

Unlike other SARS-CoV-2 variants, Omicron subvariants suffered from the loss of HLA-A*02-restricted peptides in comparison to the Wuhan original reference sequence, mainly in ORF1ab and in S protein (**Figure 8C**). Considering only HLA-A*02:Ig-reactive peptides derived from these two regions, the percentage of the loss of epitopes from ORF1ab was predominant in all subvariants, whereas the loss of S peptides was high in BA.1 and BA.2 (57.15% and 60% respectively) (**Figure 8C**). Studies comparing the new Omicron subvariants are still insipient and focused mainly on antibody response (59), but the loss of CD8^+^ T-cell epitopes has already been identified for BA.1, BA.2, and BA.3 (60).

We can infer that the minor changes observed for BA.3 against the Wuhan sequence and the limited loss of HLA-A*02:Ig reactive peptides can be associated with its limited transmission compared to BA.2 and BA.1 (59). However, additional studies regarding specific immune responses against Omicron subvariant are needed to confirm these observations.

Cytokine storm is a crucial aspect of SARS-CoV-2 infection reported for severe cases of COVID-19 (61). In that sense, we evaluated whether HLA-A*02-restricted peptides predicted for SARS-CoV-2 were biased towards the induction of IFN-γ (Th1), or IL-4 (Th2). Our findings using in silico tools showed a clear tendency for the induction of IL-4 (**Figure 6**). Such results were observed not only for all proteins of SARS-CoV-2 Wuhan original reference strain and in its five VOCs (except for ORF8 in the Alpha variant), but also for other HCoVs such as HCoV-NL63, MERS-CoV, and SARS-CoV-1. This is a clear contrast regarding other viruses such as arboviruses, for which the induction of a proinflammatory antiviral profile is predominant (20). High levels of IL-4 have been correlated to a delay in viral clearance of SARS-CoV-2 (62), however, low levels of this cytokine were also associated with long COVID-19 (63), accentuating the importance of a balance between Th1/Th2 axis for the establishment of an effective immune response against SARS-CoV-2.

A small cluster of highly hydrophobic peptides was identified as potential IFN-γ inducers in the evaluation of physicochemical parameters. These sequences presented medium to low potential of induction for IL-4 and were derived from different SARS-CoV-2 proteins (**Figure 7**). Considering that hydrophobicity is a key factor not only for the induction of IFN-γ but also for the induction of CTL immune response (64), these peptides are promising targets for cellular SARS-CoV-2-specific immune response.

Overall, the present work not only provided a comprehensive characterization of HLA-A*02-restricted peptides signatures derived from HCoV, but also identified regions of loss and gain of CD8^+^T cell epitopes in Alpha, Beta, Delta, Gamma, and Omicron variants, using the SARS-CoV-2 Wuhan original sequence as reference. Our results provide helpful insights into virus evolution, which ultimately may reflect upon the development of new therapeutic approaches and new immunization platforms aiming at controlling SARS-CoV-2 transmissions.

## Data availability statement

The original contributions presented in the study are included in the article/**Supplementary Material**, further inquiries can be directed to the corresponding author/s.

## Author contributions

Designing research study: JC-d-R. Conducting experiments and Analyzing data: AL-R, PO, HR, JC-d-R. Advisory Committee: FF, EB-S, MT. Provided reagents and funding: FF, EB-S, MT, JC-d-R. Supervised research project and acquired funding: JC-d-R. Writing the manuscript: AL-R, JC-d-R. All authors reviewed and approved the final version.
