# Supplementary material for: Surveillance of SARS-CoV-2 immunogenicity: loss of immunodominant HLA-A*02-restricted epitopes that activate CD8+ T cells
Source: Front Immunol. 2023 Nov 3;14:1229712. doi: 10.3389/fimmu.2023.1229712 (PMC10656734; doi:10.3389/fimmu.2023.1229712)
Supplement: Supplementary file 1 [file Presentation_1.pptx]

## Slide 1
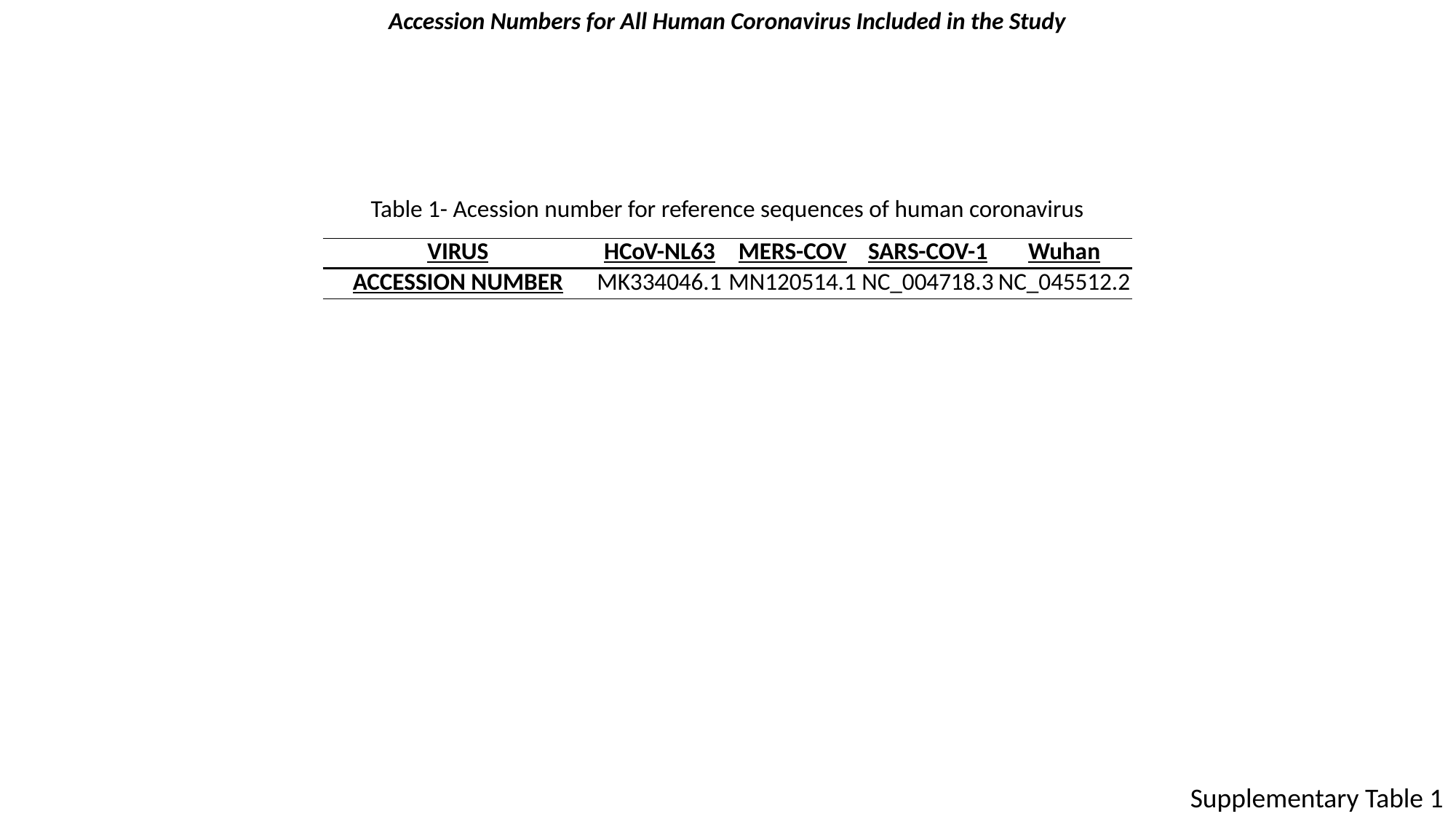

Accession Numbers for All Human Coronavirus Included in the Study
Table 1- Acession number for reference sequences of human coronavirus
| VIRUS | HCoV-NL63 | MERS-COV | SARS-COV-1 | Wuhan |
| --- | --- | --- | --- | --- |
| ACCESSION NUMBER | MK334046.1 | MN120514.1 | NC\_004718.3 | NC\_045512.2 |
Supplementary Table 1

## Slide 2
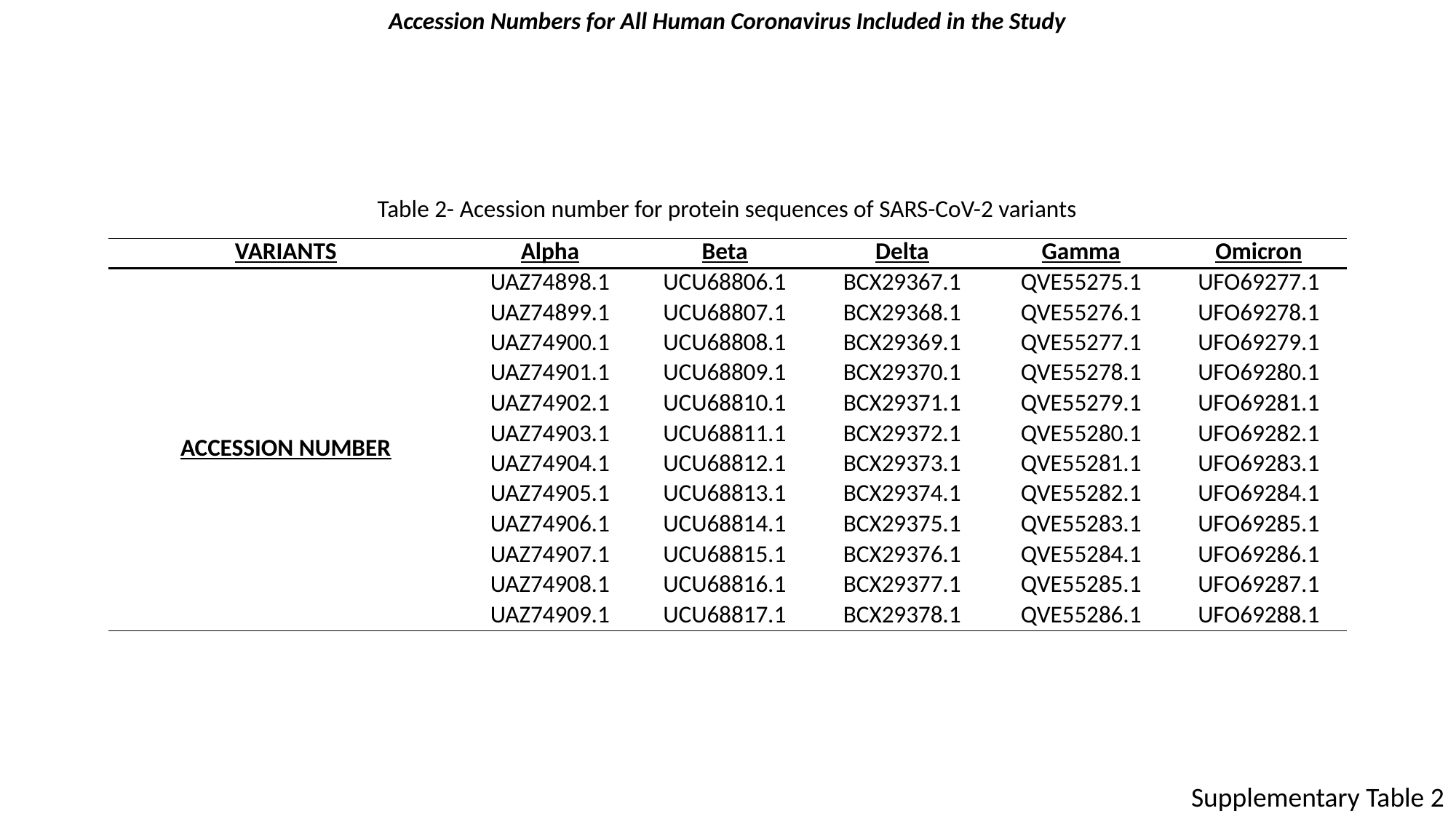

Accession Numbers for All Human Coronavirus Included in the Study
Table 2- Acession number for protein sequences of SARS-CoV-2 variants
| VARIANTS | Alpha | Beta | Delta | Gamma | Omicron |
| --- | --- | --- | --- | --- | --- |
| ACCESSION NUMBER | UAZ74898.1 | UCU68806.1 | BCX29367.1 | QVE55275.1 | UFO69277.1 |
| | UAZ74899.1 | UCU68807.1 | BCX29368.1 | QVE55276.1 | UFO69278.1 |
| | UAZ74900.1 | UCU68808.1 | BCX29369.1 | QVE55277.1 | UFO69279.1 |
| | UAZ74901.1 | UCU68809.1 | BCX29370.1 | QVE55278.1 | UFO69280.1 |
| | UAZ74902.1 | UCU68810.1 | BCX29371.1 | QVE55279.1 | UFO69281.1 |
| | UAZ74903.1 | UCU68811.1 | BCX29372.1 | QVE55280.1 | UFO69282.1 |
| | UAZ74904.1 | UCU68812.1 | BCX29373.1 | QVE55281.1 | UFO69283.1 |
| | UAZ74905.1 | UCU68813.1 | BCX29374.1 | QVE55282.1 | UFO69284.1 |
| | UAZ74906.1 | UCU68814.1 | BCX29375.1 | QVE55283.1 | UFO69285.1 |
| | UAZ74907.1 | UCU68815.1 | BCX29376.1 | QVE55284.1 | UFO69286.1 |
| | UAZ74908.1 | UCU68816.1 | BCX29377.1 | QVE55285.1 | UFO69287.1 |
| | UAZ74909.1 | UCU68817.1 | BCX29378.1 | QVE55286.1 | UFO69288.1 |
Supplementary Table 2

## Slide 3
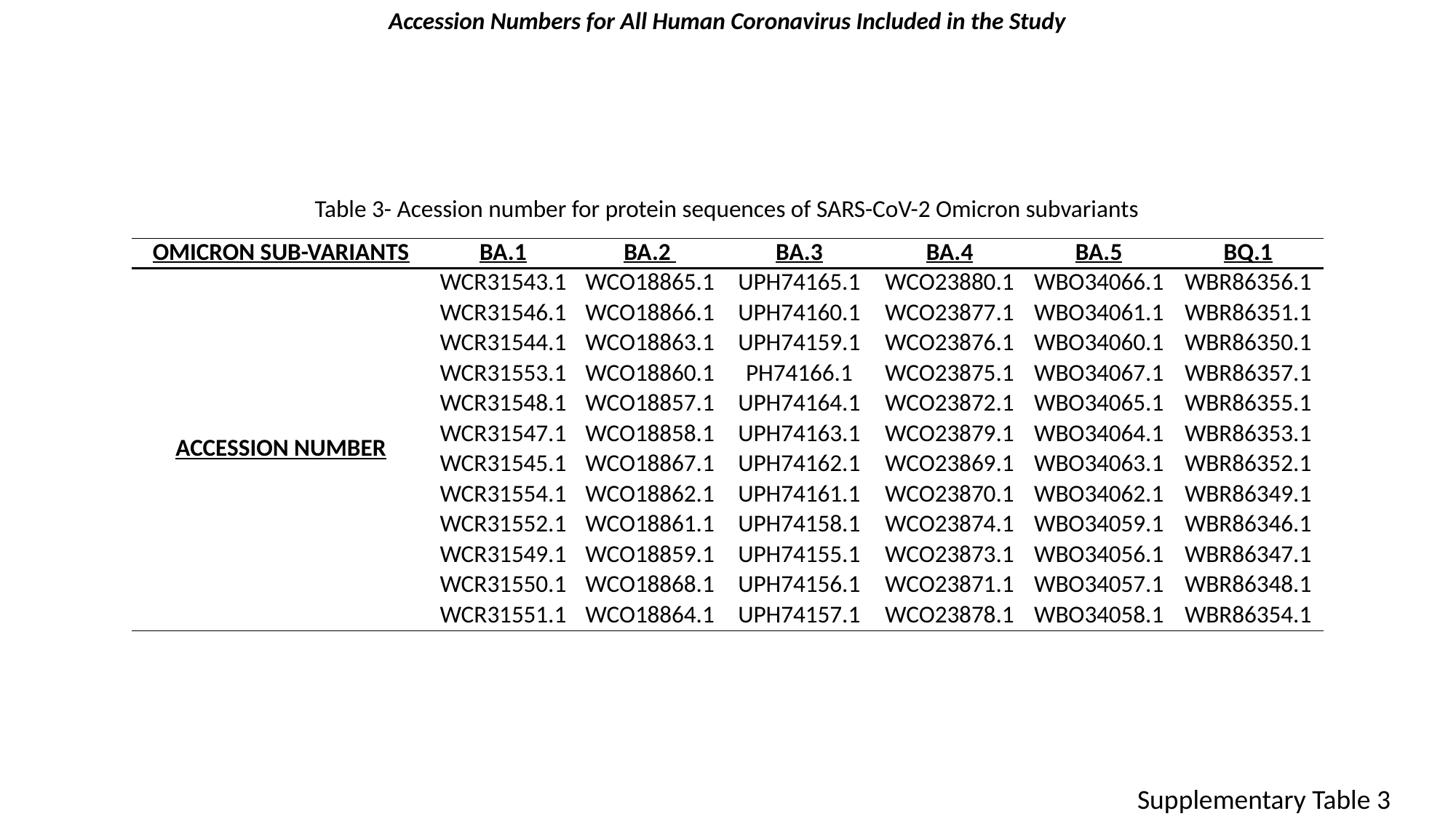

Accession Numbers for All Human Coronavirus Included in the Study
Table 3- Acession number for protein sequences of SARS-CoV-2 Omicron subvariants
| OMICRON SUB-VARIANTS | BA.1 | BA.2 | BA.3 | BA.4 | BA.5 | BQ.1 |
| --- | --- | --- | --- | --- | --- | --- |
| ACCESSION NUMBER | WCR31543.1 | WCO18865.1 | UPH74165.1 | WCO23880.1 | WBO34066.1 | WBR86356.1 |
| | WCR31546.1 | WCO18866.1 | UPH74160.1 | WCO23877.1 | WBO34061.1 | WBR86351.1 |
| | WCR31544.1 | WCO18863.1 | UPH74159.1 | WCO23876.1 | WBO34060.1 | WBR86350.1 |
| | WCR31553.1 | WCO18860.1 | PH74166.1 | WCO23875.1 | WBO34067.1 | WBR86357.1 |
| | WCR31548.1 | WCO18857.1 | UPH74164.1 | WCO23872.1 | WBO34065.1 | WBR86355.1 |
| | WCR31547.1 | WCO18858.1 | UPH74163.1 | WCO23879.1 | WBO34064.1 | WBR86353.1 |
| | WCR31545.1 | WCO18867.1 | UPH74162.1 | WCO23869.1 | WBO34063.1 | WBR86352.1 |
| | WCR31554.1 | WCO18862.1 | UPH74161.1 | WCO23870.1 | WBO34062.1 | WBR86349.1 |
| | WCR31552.1 | WCO18861.1 | UPH74158.1 | WCO23874.1 | WBO34059.1 | WBR86346.1 |
| | WCR31549.1 | WCO18859.1 | UPH74155.1 | WCO23873.1 | WBO34056.1 | WBR86347.1 |
| | WCR31550.1 | WCO18868.1 | UPH74156.1 | WCO23871.1 | WBO34057.1 | WBR86348.1 |
| | WCR31551.1 | WCO18864.1 | UPH74157.1 | WCO23878.1 | WBO34058.1 | WBR86354.1 |
Supplementary Table 3
